# Precision Medicine in Chronic Rhinosinusitis with Nasal Polyps

**DOI:** 10.1007/s11882-018-0776-8

**Published:** 2018-03-24

**Authors:** Klementina Avdeeva, Wytske Fokkens

**Affiliations:** Department of Otorhinolaryngology, Academic Medical Center, Amsterdam, The Netherlands

**Keywords:** Rhinitis, Rhinosinusitis, Nasal polyps, Treatment, Endotype, Phenotype

## Abstract

**Purpose of Review:**

Chronic rhinosinusitis is a disease with high prevalence, significant impact on health-related quality of life (HRQoL) and it is associated with substantial healthcare and productivity costs. We face an urgent need to improve the level of disease control and achieve higher patient satisfaction and disease prevention. Precision medicine is increasingly recognized as the way forward in optimal patient care. The combination of personalized care, prevention of disease, prediction of success of treatment, and participation of the patient in the elaboration of the treatment plan is expected to guarantee the best possible therapeutic approach for individuals suffering from a chronic disabling condition.

**Recent Findings:**

This is a narrative review on the current state of endotypes, biomarkers, and targeted treatments in chronic inflammatory conditions of the nose and paranasal sinuses. Different phenotypes of rhinitis and chronic rhinosinusitis (CRS) have been described based on symptom severity and duration, atopy status, level of control, comorbidities, and presence or absence of nasal polyps in CRS. The underlying pathophysiological mechanisms are diverse, with different endotypes being recognized. Novel emerging therapies are targeting specific pathophysiological pathways or endotypes. This endotype-driven treatment approach requires careful selection of the patient population who might benefit from a specific treatment.

**Summary:**

This review provides a comprehensive overview of the current state of endotypes, biomarkers and targeted treatments in chronic inflammatory conditions of the nose and paranasal sinuses.

## Introduction Including Epidemiology

Chronic rhinosinusitis (CRS) is defined as an inflammation of the nose and paranasal sinuses, characterized by two or more symptoms, one of which should be nasal blockage or nasal discharge and/or facial pain or pressure and/or reduction or loss of smell [1]. The diagnosis must be confirmed by endoscopic (nasal polyps/mucopurulent discharge/edema in the middle meatus) or radiological (mucosal changes in the sinuses/ostiomeatal complex on the CT) signs [1]. CRS affects about 11–12% of the population [2–4] although there are significant geographical differences [2]. CRS has a significant impact on health-related quality of life (HRQoL) [5–8] and is associated with substantial healthcare [9–11] and productivity costs [12].

## Phenotypes of CRS

In phenotyping of CRS the crucial point is the presence (CRSwNP) or absence (CRSsNP) of polyps according to endoscopic examination or radiological imaging [1].

Other phenotypes include CRS with aspirin-exacerbated respiratory disease (AERD) [13], allergic fungal rhinosinusitis (AFRS) [14], infectious CRS, CRS in patients with cystic fibrosis (CF) [15], and other rare phenotypes such as CRS with primary cilia dyskinesia or CRS in immune deficient patients, all of which can present as CRSwNP or CRSsNP. Moreover, clinicians treating CRS use all sorts of other factors potentially relevant in phenotyping their patients like age, gender, smoking, occupation, and presence of asthma or atopy. Recently, papers appeared using unsupervised clustering methods to identify phenotypic subgroups [21, 22]. In these studies, the outcome of relevant factors was very different with the Nakayama study giving symptoms, perennial allergy, disease severity, asthma/eosinophilic mucin, and eosinophilic inflammation as important factors and Soler stating that clustering was mainly determined by age, severity of patient reported outcome measures, depression, and fibromyalgia and indicating that traditional clinical measures, including polyp/atopic status, prior surgery, smell, and asthma did not vary among clusters [21, 22]. These differences might be explained by the different factors evaluated being only 16 in the Nakayama paper against 103 in the Soler paper. Using discriminant analysis in order to identify those measures which best separate patients into clusters, age, productivity loss, and total SNOT-22 were the most discriminating factors and a simplified algorithm based upon these three factors a predicted clustering with 89% accuracy [22]. We need more cluster analyses on clinical recognizable factors in different populations to see whether this algorithm holds and can be used in daily practice to decide on the management approach of our patients.

**Table 1:** Relevant studies in CRSwNP

Target	Drug name	Status of approval	Relevant studies in CRSwNP				
			Author	Study design	Subjects	Outcomes	Adverse events
IgE	Omalizumab	Approved by FDA for treatment of severe allergic asthma. Under investigation for use in allergic rhinitis and CRS.	Pinto et al. 2010 [32]	Randomized, placebo-controlled, double-blind study. Duration of trial—6 months. Subcutaneous injection of omalizumab (0.016 mg/kg per IU total serum IgE/ml) or placebo.	Adults with CRS with total serum IGE between 30 and 700 IU/ml. *N* = 14 (treatment group = 7, placebo group = 7).	No significant changes on CT scan evaluation (*P* < 0.391), in QoL (*P* < 0.60), in olfaction (*P* < 0.31), endoscopy scores (*P* < 0.58), in eosinophils in nasal lavage (*P* < 0.47), NPNIF (*P* < 0.31), sinonasal symptoms (*P* < 0.21). Trend towards less use of rescue medications in treatment group.	No side effects during the study.
			Gevaert et al. 2013 [33]	Randomized, double-blind, placebo-controlled, 2-center study. Duration of trial—20 weeks. Subcutaneous injection of omalizumab every 2 weeks (8 injections in total) or every month (4 injections in total) based on total IgE levels and body weight (max dose 375 mg) or placebo.	Adults with CRSwNP and comorbid asthma for more than 2 years. *N* = 24 (treatment group = 16 subjects, placebo group = 8 subjects).	Significant reduction in the treatment group: polyp size (*P* = 0.02), Lund-Mackay score (*P* = 0.04), nasal congestion (*P* = 0.002), anterior rhinorrhea (*P* = 0.003), loss of sense of smell (*P* = 0.004), wheeze (*P* = 0.02), dyspnea (*P* = 0.02). Cough and spirometric results did not reach significant differences. Mental health did not improve significantly. Physical health (*P* = 0.02), sleep (*P* = 0.03), general symptoms (*P* = 0.01), AQLQ (*P* = 0.003), activity limitations (*P* = 0.002), symptoms (*P* = 0.01) and emotional function (*P* = 0.02) significantly improved in treatment group).	Common cold appeared significantly more often in the treatment group (*P* = 0.02). Asthma attack (*n* = 1, placebo group). Fatal lymphoblastic lymphoma 1 year after finishing the study (*n* = 1, treatment group).
	Ligelizumab		No trials for CRSwNP yet.				
IL-5	Mepolizumab	Approved by FDA for treatment of severe eosinophilic asthma.	Bachert et al. 2017 [43]	Randomized, double-blind, placebo-controlled, multicenter study. Duration of trial—25 weeks. Intravenous infusion of 750 mg mepolizumab or placebo every 4 weeks, 6 doses.	Adult patients with severe recurrent bilateral polyposis who required surgery. *N* = 105 (treatment = 54, placebo = 51).	Mepolizumab significantly reduced the number of patients who needed surgery (*P* = 0.006), improved VAS score of nasal polyposis (*P* = 0.001), endoscopic polyp score (*P* = 0.031), mean individual symptom VAS scores (rhinorrhea, mucus in the throat, nasal blockage, loss of smell), SNOT-22 scores, PNIF (*P* = 0.026). No significant differences between mepolizumab in EQ-5D index scores, olfaction, lung function.	AEs more frequent in placebo group: headache, nasopharyngitis. AEs more frequent in treatment group: oropharyngeal pain, back pain, influenza, pyrexia.
	Reslizumab	Approved by FDA for treatment of severe eosinophilic asthma.	Gevaert et al. 2006 [42]	Phase I. single-dose, randomized, double-blind, placebo-controlled, 3-arm, parallel-group, 2-center safety and pharmacokinetic study. Duration of the trial—36 weeks. Intravenous infusion of reslizumab 3 mg/kg or 1 mg/kg or placebo.	Adult patients with massive bilateral nasal polyposis or recurrent nasal polyposis after surgery. *N* = 24	(Study was not designed and powered to detect treatment differences in efficacy variables.) No significant differences in symptom scores or in nasal peak inspiratory flow values. Significant decrease in blood eosinophil counts in both treatment groups. Significant suppression of nasal IL-5.	AEs: upper respiratory tract infection (treatment groups = 5 each, placebo group = 4). No major differences in other AEs.
IL-5Rα receptor	Benralizumab	Approved by FDA for treatment of severe eosinophilic asthma.	No trials for CRSwNP yet.				
Anti-IL-4/IL-13	Dupilumab	Approved by FDA for treatment of eczema.	Bachert et al. 2016 [50]	Randomized, double-blind, placebo-controlled parallel-group study. Duration of the trial—32 weeks. Subcutaneous dupilumab (loading dose 600 mg followed by 15 weekly doses of 300 mg) and placebo.	Adult patients with bilateral nasal polyposis and chronic symptoms of sinusitis despite intranasal corticosteroid treatment for at least 2 months. *N* = 60 (30 patients in each group).	Significant improvement in the treatment group in polyp score (*P* < 0.001), Lund-Mackay CT score (*P* < 0.001), peak inspiratory flow (*P* = 0.002), SNOT-22 (*P* < 0.001).	AEs: 25 of 30 patients in placebo group and 30 of 30 patients in the treatment group. AEs: mild-to-moderate nasopharyngitis, injection site reactions, headache. No serious adverse events were considered to be related to dupilumab.
	Tralokinumab	In phase III trials for severe asthma (unsatisfactory results of STRATOS 2 and TROPOS trials).	No trials for CRSwNP yet.				
	Lebrikizumab	In phase II for atopic dermatitis, idiopathic pulmonary fibrosis. Discontinued for asthma, COPD, Hodgkin’s disease.	No trials for CRSwNP yet.				
Siglec-8		Phase II trial is underway.	No trials for CRSwNP yet.				
CRTh2/DRP			No trials for CRSwNP yet.				
CXCR2 receptors			No trials for CRSwNP yet.				
IL-17A	Brodalumab	Approved by FDA for treatment patients with moderate-to-severe plaque psoriasis.	No trials for CRSwNP yet.				

## Endotyping CRS

To implement precision medicine, a shift in approach strategy, i.e., from phenotyping towards endotyping, is needed. Endotype classification is based on underlying pathophysiological mechanism. Different phenotypes in CRS can show a very similar endotype and vice versa, e.g., CRSwNP in N-ERD [23] and CF have very similar phenotypes but very different endotypes.

In Europe and the USA, the most prevalent endotype in CRSwNP shows a type 2 inflammatory response and is characterized by high prevalence of eosinophils, mast cells, and basophils, as well as elevated type 2 cytokines (IL-4, IL-5, IL-9, IL-13, IL-25, and IL-33) and Th2 cells.

Eosinophilia is induced by IL-5, IL-25, and IL-33. Local and systemic IgE production takes place in allergic patients with the involvement of IL-4 and IL-13 [24, 25].

Recently, a cluster analysis of biomarkers of inflammation in CRS was performed in European patients resulting in 10 clusters, of which 3 clusters with low or undetectable IL-5 and IL-17, eosinophilic cationic protein, IgE, and albumin concentrations, 1 cluster with low IL-5 but high IL-17, 3 clusters with intermediate values of IL-5 and IgE, and 3 clusters with high concentrations of those markers. The first group of IL-5-negative clusters clinically resembled a predominant chronic rhinosinusitis without nasal polyp (CRSsNP) phenotype without increased asthma prevalence, the 4 clusters in the middle showed a mixed CRSsNP/CRSwNP phenotype and increased asthma phenotype and the IL-5-high clusters an almost exclusive nasal polyp phenotype with strongly increased asthma prevalence [26]. A Chinese group using 18 variables resulted in 5 clusters with different polyp recurrence rates, based on the presence of predominantly plasma cells, lymphocytes, neutrophils, eosinophils, or mixed inflammatory cells in polyps [27]. Multiple biological agents targeting type 2 inflammation have been developed and studied for disease control of eosinophilic asthma and are potential therapeutic candidates for CRSwNP.

Non-Th2 inflammation is mainly characterized by the presence of neutrophils, elevated type 1 cytokines (interferon-γ), and Th1 cells, which can be triggered by infection or chronic irritation, such as air pollution.

Type 1 immune response may contribute to disease chronicity and is more common in CRSsNP [25••, 28].

Eosinophils are also common in CRSsNP, although in much lower concentrations [29].

## Precision Medicine in CRSwNP

Precision medicine is a medical model aiming at the customization of healthcare—with medical decisions, practices, and/or products tailored to the individual patient. Based on the knowledge of mechanisms of the disease, precision medicine generally combines diagnosis and treatment to select optimal management [30]. Patient participation in the decision of the treatment plan, prediction of success of the initiated treatment, strategies to prevent progression of disease, and personalized endotype-driven treatment are the cornerstones of precision medicine [31**••**]. For endotype-driven treatment, adequate and standardized way of endotyping and insight into the biomarkers predicting the success of treatment are crucial.

## New Treatment Options in CRSwNP

The primary modality for treatment of CRSwNP is pharmacological and consists of systemic and/or topical glucocorticosteriods [32] and saline irrigations and occasionally antibiotics. If conservative measures fail, surgery is performed and then medical therapy is continued. Here, we discuss new treatment options based on specific control of the Th2 inflammatory endotype most common in CRSwNP that have already shown efficacy in randomized controlled trials and potential options in the near future and are summarized in Table 1 [33, 34].

### Anti-IgE

Omalizumab is a recombinant humanized anti-IgE monoclonal antibody that proved efficacy in patients with severe allergic asthma [35, 36]. The mechanism of action involves selective binding to free circulating IgE, which decreases the expression of IgE receptors on mast cells, basophils, and dendritic cells and thereby interferes with activation of these effector cells. Omalizumab is approved by the European and US regulatory authorities for the treatment of severe allergic asthma and is currently under investigation for its use in the treatment of allergic rhinitis and CRS.

The first trial with omalizumab in CRSwNP was negative stating that omalizumab had a small and clinically irrelevant effect on CRS [16]. This trial however was underpowered and patients with CRSsNP and CRSwNP were combined. This emphasizes the importance of endotyping to select patients who will benefit from anti-IgE treatment.

In CRSwNP patients with comorbid asthma, omalizumab showed reduction of nasal symptoms and improved quality of life, reduced nasal endoscopic polyp scores and CT Lund-Mackay scores, and reduced need for further medical or surgical treatment [17].

New promising biologicals targeting IgE have been developed with the aim of improving anti-IgE treatment. Ligelizumab is a monoclonal antibody, which, compared to omalizumab, shows higher affinity and increased suppression of free IgE [37] and greater efficacy on inhaled skin allergen responses in a small study in patients with mild allergic asthma [38].

Quilizumab is a humanized monoclonal antibody targeting specifically the M1 prime epitope of membrane IgE that prevents IgE production in humans [39]. However, targeting the IgE pathway via depletion of IgE-switched and memory B cells was not sufficient for a clinically meaningful benefit for adults with allergic asthma uncontrolled by standard therapy [40].

### Anti-IL-5

In a large subset of patients with CRSwNP, the presence of tissue eosinophilia is related to IL-5 [41]. T cells and ILC2 are the most likely source of IL-5 and anti-IL-5 treatment may reduce eosinophil-related inflammation and polyp size [42].

IL-5 is responsible for survival, maturation, and activation of eosinophils at the bone marrow and the site of inflammation [43] and is a key mediator in type 2 eosinophilic inflammation [41].

To interfere with the IL-5 pathway, novel biologicals are developed targeting IL-5 and its receptor IL-5Rα on the effector cells. Mepolizumab and reslizumab are both humanized anti-IL5 mAb that neutralize IL-5. Both biologicals are approved by the European and US Food and Drug Association (FDA) for its use in the treatment of severe eosinophilic asthma [44].

A phase II trial showed that one single intravenous injection of reslizumab significantly reduced blood eosinophil counts and nasal IL-5 levels in patients with CRSwNP and improved nasal polyp scores in half the patients for up to 4 weeks with a better response in patients with baseline increased IL-5 levels [19].

Recently, a randomized, double-blind, placebo-controlled trial with 750 mg of intravenous mepolizumab or placebo every 4 weeks for a total of six doses in addition to daily topical corticosteroid treatment in patients with CRSwNP with recurrent nasal polyposis requiring surgery showed a significant reduction in the number of patients needing surgery. There was also a significant improvement in nasal polyposis severity VAS score, endoscopic nasal polyp score, all individual VAS symptom scores, and SNOT score in the mepolizumab-treated groups compared with placebo groups. Mepolizumab’s safety profile was comparable with that of placebo [18••].

In asthma, cluster analysis has shown three predictors in four primary clusters to be related to better response to mepolizumab: blood eosinophils, airway reversibility, and body mass index. The reduction in exacerbations was significantly greater in patients who received mepolizumab (clusters 2, 3, and 4) with raised eosinophils (responder population). Cluster 2 with low airway reversibility (mean, 11%) had a 53% reduction in exacerbations. These patients more frequently reported sinusitis and nasal polyposis. Those with higher airway reversibility (mean, 28%) were further split by body mass index. The non-obese versus obese (clusters 3 and 4) had a 35 and 67% reduction in exacerbations, respectively [45]. In CRSwNP, it has until now not been possible to identify which patients react most favorably to anti-IL-5 treatment.

Benralizumab is a humanized mAb against the highly expressed IL-5Rα receptor on eosinophils. Its efficacy and safety in uncontrolled asthma with eosinophilia has been demonstrated in a phase III trial [46]. So far, no studies are published on its use in upper airway diseases.

### Anti-IL-4/IL-13

IL-4 and IL-13 share the same receptors: a type 1 receptor with the IL-4Rα subunit that can only be activated by IL-4, and the type 2 receptor, a heterodimer consisting of IL-4Rα and IL-13Rα1, that can be activated by both IL-4 and IL-13. This explains their mutual and important role in the type 2 inflammation.

Dupilumab is a fully human monoclonal antibody that blocks the IL-4Rα subunit and first was shown to improve clinical responses in adults with moderate-to-severe atopic dermatitis in a dose-dependent manner [47, 48]. It is approved for the treatment of patients with moderate-to-severe atopic dermatitis in the European Union and the USA. It has also been shown to increase lung function and reduce severe exacerbations in patients with uncontrolled persistent asthma despite medium-to-high dose of inhaled corticosteroids and a long-acting β2-agonist [49, 50].

Recently, a study was published in patients with CRSwNP showing a positive effect of dupilumab on nasal polyp score, CT score, 22-item SinoNasal Outcome Test, and sense of smell in patients with CRSwNP refractory to topical corticosteroids [20].

Interestingly, two anti-IL-13 only monoclonal antibodies tralokinumab and lebrikizumab do not seem to fulfill their promise in asthma trials.

## Other Type 2 Directed Therapeutical Options

### Siglec 8

Siglecs (sialic acid immunoglobulin-like lectins) are cell surface proteins found predominantly on cells of the immune system. Among them, Siglec-8 is uniquely expressed by human eosinophils and mast cells as well as basophils. When this structure is engaged with antibodies or glycan ligands, eosinophils undergo apoptosis and release of preformed and newly generated mediators from human mast cells is inhibited without affecting their survival [51].

Siglec-8 is one of the possible targets for biological treatment of eosinophil and mast cell-related diseases such as asthma and CRSwNP. A phase II trial with a therapeutic antibody that targets Siglec-8 in patients with CRSwNP is underway.

### CRTh2/DRP-Antagonist

Chemoattractant receptor-homologous molecule expressed on TH2 cells (CRTH2) and D-type prostanoid receptor (DPR) are G-protein-coupled prostaglandin (PGD2) receptors.

Two CRTH2/DRP antagonists are also currently under clinical investigation and may be especially interesting in aspirin-exacerbated respiratory disease [52].

### Therapies Targeting Non-type 2 Inflammation

Development of novel therapies targeting non-type 2 inflammation seems more challenging than in type 2 inflammation. Different biologicals have been investigated but only few showed little efficacy.

CXC chemokine 2 receptor (CXCR2) antagonists target the CXCR2 receptors on neutrophils and prevent their activation through the chemokine IL-8. Their efficacy has been investigated in the treatment of severe asthma, in which no clinical improvement was seen, despite reduction of neutrophils in sputum and blood [53, 54].

Brodalumab is a human anti-IL17A mAb designed to target IL-17A, a cytokine that is associated with neutrophilic inflammation and corticosteroid resistance. A trial of brodalumab in patients with uncontrolled moderate-to-severe asthma, without being selected for neutrophilic inflammation, reported no improvement of symptoms or lung function [55].

So far, no trials evaluating targeted treatments of non-type 2 inflammation have been conducted in CRSwNP. The relative poor evolution in the development of biologicals targeting non-type 2 inflammation indicates that non-type 2 inflammation still needs further untangling.

## Conclusion and Current Challenges in Precision Medicine and Endotype-Driven Treatment

Implementation of the principles of precision medicine into the management of upper airway diseases like CRSwNP is a major task for the next decade. Endotype-driven treatment is an important component of precision medicine especially in patients with uncontrolled severe disease. Monoclonal antibodies could be a potential new treatment when we can find the patients with the phenotype and endotype that will benefit most from these treatments.

The ability to predict which patients will respond favorably to a certain monoclonal antibody will be a key issue in achieving cost-effectiveness. Ideally, we should be able to discriminate these patients early in the disease and treat them early to prevent multiple surgeries in the years to follow and potentially also to prevent the development of lower airway disease.
